# Effect of fermentation on structural properties and antioxidant activity of wheat gluten by *Bacillus subtilis*

**DOI:** 10.3389/fnut.2023.1116982

**Published:** 2023-02-23

**Authors:** Peng-hui Zhao, Yin-Chen Hou, Zhen Wang, Ai-Mei Liao, Long Pan, Jie Zhang, Yu-Qi Dong, Zhe-Yuan Hu, Ji-Hong Huang, Xing-Qi Ou

**Affiliations:** ^1^Henan Provincial Key Laboratory of Biological Processing and Nutritional Function of Wheat, College of Biological Engineering, Henan University of Technology, Zhengzhou, China; ^2^State Key Laboratory of Crop Stress Adaptation and Improvement, College of Agriculture, Henan University, Kaifeng, China; ^3^School of Food and Pharmacy, Xuchang University, Xuchang, China; ^4^College of Life Science and Technology, Henan Institute of Science and Technology, Xinxiang, China

**Keywords:** gluten, *Bacillus subtilis*, fermentation, antioxidant activity, structural characterization

## Abstract

*Bacillus subtilis* has been extensively studied for its ability to inhibit the growth of harmful microorganisms and its high protease activity. In this study, *Bacillus subtilis* was used to ferment gluten and assess the effects of the fermentation process on the physicochemical, microstructure and antioxidant properties of gluten. The results of Fourier infrared spectroscopy (FT-IR) and circular chromatography (CD) showed a significant decrease in the content of α-helix structures and a significant increase in the content of β-sheet structures in gluten after fermentation (*p* < 0.05). Sodium dodecyl sulfate-polyacrylamide gel electrophoresis (SDS-PAGE) showed that glutenin was degraded into small molecular peptides with a molecular weight of less than 26 kDa after 24 h of fermentation; meanwhile, the fermentation process significantly increased the free amino acid content of the samples (*p* < 0.05), reaching 1923.38 μg/mL at 120 h of fermentation, which was 39.46 times higher than that at 24 h of fermentation (*p* < 0.05). In addition, the fermented back gluten has higher free radical scavenging activity and iron reduction capacity. Therefore, fermented gluten may be used as a functional food to alleviate oxidative stress. This study provides a reference for the high-value application of gluten.

## 1. Introduction

Wheat gluten (WG) is a by-product of wheat starch production. WG is mainly composed of wheat gluten and wheat alcoholic soluble protein with a relatively complete amino acid composition, making it a nutritious and inexpensive source of plant-based protein. The lack of hydrophilic amino acid residues in gluten protein results in a poor water solubility of gluten protein, which limits its application to a large extent (1). Fermentation technology is considered to be one of the most effective means of modifying the properties of gluten; first, fermentation is a biomodification pathway in which microorganisms produce a variety of enzymes through their own metabolism to break down and convert the product into more absorbable nutrient factors and bioactive small molecule peptides (2); second, fermentation process is thought to improve the digestibility and absorption of nutrients in living organisms and has the potential to increase antioxidant activity (3). In recent years, with the rapid development of microbial fermentation technology, great progress has been made in improving the nutrition of fermented grains; in short, fermentation can improve the nutritional and functional quality of grains, as well as the efficiency of grain by-product utilization (4).

*Bacillus subtilis* is a Gram-positive bacterium found in soil and is widely used in biological fermentation processes in the food industry. *Bacillus subtilis* has the ability to produce antimicrobial proteins and a variety of protease activities such as antifungal proteins (5, 6), nattokinase (7), pectinase (8) and various bioactive peptides such as antimicrobial peptides and antioxidant peptides etc. (9–11). It is a beneficial microbial strain with potential applications in biotechnology, advanced materials (12), agriculture (13), animal husbandry (14), food research and development (15) and biomedicine (16).

In recent years, *in vitro* and *in vivo* studies on the effect of *Bacillus subtilis* fermentation on enhancing the antioxidant activity of cereals have been reported repeatedly. However, to our knowledge, the fermentation substrates in current Bacillus subtilis fermentation-related studies have mostly used legumes, maize, and other plant raw materials (17–19), and there are few reports on changes in the hydrolysis and antioxidant properties of cereal prions after *Bacillus subtilis* fermentation. In this study, *Bacillus subtilis* was used to ferment WG and the fermentation products were analyzed for amino acids, structures were determined and the antioxidant activity and thermal properties of gluten after fermentation were examined. This study may provide the necessary data for the development of functional gluten products.

## 2. Materials and methods

### 2.1. Materials and microorganism

Wheat gluten obtained from ‘Bainong 207,’ Xinxiang Nongle Seed Industry Co., Ltd. (Xinxiang, China). *Bacillus subtilis* B53 is a laboratory preservation strain (Henan Province Industrial microbial species Preservation Center). SDS-PAGE kit was purchased from Solarbio (Wuhan, China). BCA protein assay kit was obtained from Glpbio (lot no. GK10009, Shanghai, China). All aqueous solutions were prepared with ultrapure water. All the chemicals used in this study were of analytical grade.

### 2.2. Preparation of fermented wheat gluten

Fermented wheat gluten was prepared according to the method of Zhao et al. (20) with modifications. Briefly, the activated *Bacillus subtilis* was inoculated in LB medium and incubated for 24 h at 37°C to prepare the seed solution. Ten g of WG was taken, 100 mL of distilled water was added at a solid–liquid ratio of 1/10 (w/v), sterilized at 121°C for 15 min, and each bottle was inoculated according to the set inoculation conditions, and the fermentation was carried out at 37°C in a shaker at 150 rpm.

Fermentation supernatant of 24, 72, 120, and 168 h were taken respectively, the fermentation broth of the strain was centrifuged at 4,000 × *g* for 20 min to remove the precipitated bacteria and unfermented gluten residue. The supernatant was filtered through a 0.45 μm membrane and the filtered supernatant was freeze-dried to give the fermented wheat gluten (FWG) samples.

### 2.3. Morphology of FWG

The morphology of the samples was observed using a Zeiss EVO LS-15 scanning electron microscope with reference to the method of Chen et al. (21) with slight modifications. The accelerating voltage was 5 kV. Samples were uniformly sprayed with 15 nm gold foil on the surface using an ion ejector before observation.

### 2.4. Secondary structure characterization of FWG

The secondary structure of FWG was characterized by a CD at different fermentation times. The ellipticity (medg) data in the range of 190–260 nm were recorded at a scanning speed of 60 nm/min. Secondary structure content has CDPro program software calculations.

According to the method of Liao (22), FWG samples with different times of fermentation and KBr were mixed well at a ratio of 1:100 (w/w) and then well ground. Nicolet iS20 (Thermo Scientific) was used for FT-IR characterization of FWG. The mixture was added to the test bench under dry air and room temperature conditions to achieve a uniform thickness. The parameters were set as follows: scan range of 4,000–400 cm^−1^, 32 scans, resolution of 0.25 cm^−1^, and signal acquisition. The results were analyzed and plotted using Origin 8.0 software.

### 2.5. Analysis of the free amino acid composition

The free amino acid (FAA) composition and content of samples with different fermentation times were determined using a fully automated amino acid analyzer (Sykam, Germany), referring to the method of Liao et al. (23). The ratio of branched-chain amino acids (BCAA) to aromatic amino acid content was used as the Fischer ratio.

### 2.6. Determination of soluble protein content of FWG

The fermentation broth was collected at various fermentation times and then centrifuged at 8,000 × *g* for 15 min at 4°C. The soluble protein content of the supernatant of the extract was determined by BCA kit with bovine serum albumin as the standard. BCA was measured in a 96-well plate. Two hundred μL dye solution was added to 25 μL sample and then incubated at 37°C for 30 min, and then the absorbance of the solution was measured at 562 nm. The conversion formula of bovine serum protein standard curve is Y=0.0007X+0.1441 , $\;R^{2}=0.9987$ .

### 2.7. Sodium dodecyl sulfate-polyacrylamide gel electrophoresis

SDS-PAGE electropherograms were used to study the degradation of gluten proteins during fermentation, and the operation was referred to as the method of Lee et al. (24) with slight modifications. Gluten fermentation broth with different fermentation time intervals was taken and kept in a water bath shaker (150 r/min) for 1 h at 50°C and mixed well and then centrifuged at 8,000 × *g*, 4°C for 10 min to take the supernatant and set aside.

Prepare the gels according to the kit (4% concentrated gel and 15% separation gel). Add the supernatant proportionally to the sample buffer, then add the sample to the gel, and perform electrophoresis at 120 V. After completion, the gels were stained with 0.1% (w/v) Coomassie Brilliant Blue R-250 and scanned using Image Scanner III. A quantitative analysis of the stained protein bands was performed.

### 2.8. Antioxidant activity measurement of FWG

#### 2.8.1. DPPḤ scavenging activity

Determination of the DPPḤ radical scavenging rate of fermentation broth was referred to the literature (17) with modifications. Take 1.0 mg of FWG at different times of fermentation in a centrifuge tube, add 1.0 mL of deionized water to dissolve it fully as the solution to be measured, place 100 μL of the solution to be measured in a 96-well plate, then add 100 μL of DPPH-anhydrous ethanol solution (100 μmol/L), mix well and react for 30 min at room temperature and avoid light, measure the absorbance at 517 nm and label as A_1_; replace the solution to be measured with deionized water and measure the absorbance as A_0_; replace DPPH-anhydrous ethanol with deionized water and measure the absorbance value (A_2_).


(1) $$\mathrm{DPPH}\cdot \left(I\%\right)=\frac{A_{0}-A_{1}+A_{2}}{A_{0}}\times 100\%$$

#### 2.8.2. ·OH scavenging activity

Determination of the DPPḤ radical scavenging rate of fermentation broth with reference to literature (17). Take 1.0 mL of the above test solution, add 1.0 mL of 9 mmol/L FeSO_4_, 1.0 mL of 9 mmol/L salicylic acid and 0.5 mL of 0.1% H_2_O_2_ in order, shake well, and react in a water bath at 37°C for 30 min. Using deionized water as reference, measure the absorbance value at 510 nm as A_1_, deionized water was used to replace the solution to be measured, and the absorbance value was measured as A_0_. 1.0 mL of the solution to be measured was added with 2.5 mL of deionized water, and the absorbance value was measured as A_2_.


(2) $$\cdot \mathrm{OH}\;\left(I\%\right)=\frac{A_{0}-A_{1}+A_{2}}{A_{0}}\times 100\%$$

#### 2.8.3. ABTS scavenging activity

Determination of the ABTS radical scavenging rate of fermentation broth with reference to literature (17). The ABTS radical stock solution was prepared by mixing equal volumes of 7.4 mmol/L ABTS and 2.6 mmol/L potassium persulfate solution and reacting in the dark for 12–16 h. The solution was diluted with deionized water so that the absorbance at 734 nm was 0.70 ± 0.02. Add 0.9 mL of ABTS radical stock solution to 100 μL of the solution to be tested, mix well, and measure the OD value at 734 nm for 5 min at room temperature and avoid light as A_1_; use anhydrous ethanol instead of the solution to be tested to measure the OD value as A_0_; use deionized water instead of the ABTS radical stock solution to measure the OD value as A_2_.


(3) $$\mathrm{ABTS}\;\left(I\%\right)=\frac{A_{0}-A_{1}+A_{2}}{A_{0}}\times 100\%$$

#### 2.8.4. Iron-reducing power

Measurement of the iron-reducing power with reference to literature (25). Take 1.0 mL of the above test solution, add 2.5 mL of 0.2 mol/L PBS and 2.5 mL of 1% K_3_Fe(CN)_6_ in order, mix well and put the reaction in a water bath at 50°C for 20 min at constant temperature, then add 1.0 mL of 10% trichloroacetic acid (TCA) solution to the mixture, take 2.5 mL of the mixture, add 2.5 mL of deionized water and 1.0 mL of FeCl_3_ solution in order. Using deionized water as the zeroing solution, the OD value was measured at 700 nm, and the absorbance value of the solution to be measured at 700 nm was used to measure the magnitude of its reducing power.

### 2.9. Thermal stability analysis of FWG

Thermogravimetric analysis (TGA) of the samples was performed with reference to the method of Ma et al. (26) with minor modifications. Specifically, a sample of bran powder dried to a constant weight of about 5 mg was weighed before and after modification and spread evenly in a lead mold for pressing. Measurement conditions. The nitrogen flow rate was set to 50 mL/min, the scanning temperature range was set to 0–200°C, and the heating rate was set to 10°C/min. Three parallel tests were performed for each sample, and the results were averaged.

### 2.10. Statistical analysis

The data was obtained from the samples after three replicates of fermentation and represented by mean ± SD. Analysis of variance was performed using SPSS 20.0 software. Duncan’s multiple range test was used to determine the differences in means, and *p* < 0.05 was considered statistically significant. Origin 2018 was used to draw graphs.

## 3. Results and discussion

### 3.1. The micromorphology of FWG

**Figure 1:**
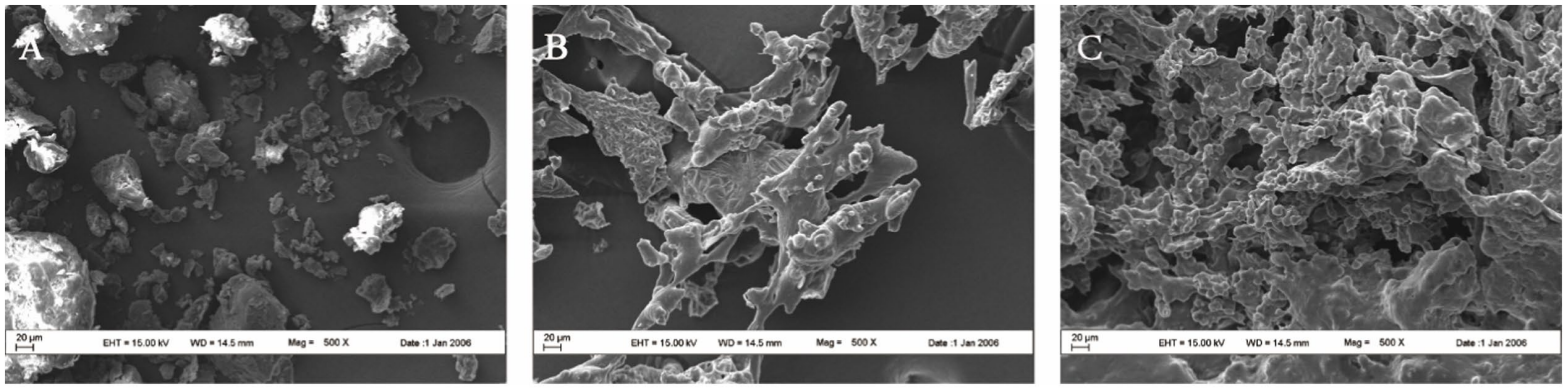
Effect of fermentation on the structure of WG. **(A)** Fermented gluten for 0 h. **(B)** Fermented gluten for 72 h. **(C)** Fermented gluten for 168 h.

The scanning results of FWG at different fermentation times are shown in Figure 1, where significant differences in the microstructure of the samples can be observed at different fermentation times. At zero time of fermentation, the samples had a large number of irregular lamellar structures with dense structures and smooth surfaces. After 72 h of fermentation, the surface of the samples was no longer smooth and porous. As the fermentation process progressed, the pores in the microstructure of the samples at 168 h gradually became dense and the surface gradually took on a fragmented appearance compared to the previous sheet. A tight 3D network structure was formed and it is hypothesized that these microstructural changes were due to proteolysis caused by proteases produced by *Bacillus subtilis* fermentation (27). The presence of a large number of pores in the microscopic samples also verified the experimental results of previous experiments in which protein degradation produced large amounts of FAA (28). According to the microstructure, the proteins in the fermentation samples are gradually degraded by the combined action of endogenous proteases and proteases secreted by microorganisms, and the molecular structure becomes stretched, exposing more easily the internal hydrophobic regions and leading to the structural depolymerization of the bran.

### 3.2. Effect of fermentation on FWG secondary structure

The gluten was characterized at different fermentation times using FT-IR and CD.

**Figure 2:**
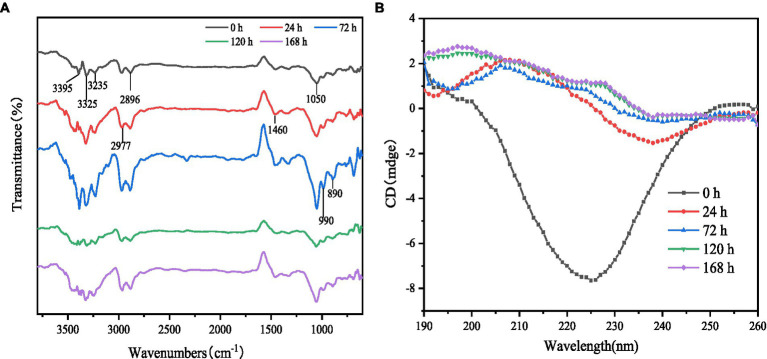
Infrared spectrum and circular dichrography of gluten at different fermentation times. **(A)** FT-IR spectra of gluten at different times of fermentation. **(B)** Circular dichroism of gluten at different times of fermentation.

FT-IR results are shown in Figure 2A. Although the location of the absorption peaks was approximately the same for the samples at different fermentation times, there were significant differences in the intensity of the absorption peaks. 3,750–3,000 cm^−1^ region was considered to have N-O and C-H stretching vibrations; compared to control, the samples after fermentation showed enhanced intensity of the absorption peaks at 3,400–3,200 cm^−1^ and 3,000–2,890 cm^−1^ for gluten, which was attributed to multimolecular conjugation in the amide region (triple and double bonds in the unsaturated carbon, C-H stretching vibrations on the benzene ring), hydrogen bonding interactions and C-H stretching vibrations on the saturated carbon, including vibrations on the aldehyde group (29, 30). 1,125–1,000 cm^−1^ reflects C-O stretching and C-O-H bending of alcohols and phenols and the presence of a strong absorption band at 1,050 cm^−1^, which is thought to be deformation of the aromatic ring C-H (31). The characteristic peak intensity gluten fermentation was followed by the highest peak intensity and peak width at 72 h of fermentation. This indicates that the protease activity is strongest at this time, when the gluten proteins are microbially degraded into small molecules and short peptides, with changes in the internal hydrogen bonding arrangement, after which the protease activity begins to decrease as fermentation progresses, with the lowest alcohol and phenol content after 120 h of fermentation. The peak intensity of gluten increased at 890, 990, and 1,050 cm^−1^ after fermentation, implying an increase in the content of olefins and aromatic substances after fermentation and an enhanced absorption of C-H out-of-plane bending vibrations, which may be related to atomic polarity and charge transfer (32).

Proteins, on the other hand, are usually chiral molecules with circular dichroism. The secondary structure of FWG determines their spectral signatures in the UV range of 190–260 nm. The differences in peak position and peak area in the spectra reflect the secondary structure nature and main chain conformation of the products. Therefore, in this study, the secondary structure of the proteins in the FWG was determined in the far UV region. The scan results are shown in Figure 2B. The CD scan showed a positive peak near 210 nm with increasing intensity with increasing fermentation time, indicating the presence of a β-sheet structure in the sample with a gradual increase in content. The positive peak at 195 nm and the negative peak near 200 nm indicated an α-helical conformation in the sample. The small, broad positive peak near 217 nm indicates the presence of an irregular coil conformation in the sample. It can be seen that the secondary structure of the FWG protein includes α-helix, β-sheet, β-loop and irregular helix structures. This is consistent with the results obtained from the CDPro V3 software analysis (courtesy of Colorado State University).

The proportions of FWG protein secondary structures were calculated using CDPro software (Table S1).

As fermentation proceeded, the α-helix structure content decreased and the β-sheet content increased significantly (*p* < 0.05); there was no significant trend in the β-turn content; the random coil content showed a trend of first decreasing and then increasing. The α-helix consists of amino acid residues of N-H forming hydrogen bonds with the amino side and three amino acid residues of C=O. Since all peptide chain peptide bonds can form hydrogen bonds, the α-helix is very stable and the hydrogen bonds between amino acid residues of the significantly reduced α-helix conformation after fermentation are disrupted and gradually exposed to irregular structure by the action of microorganisms (25, 27). This result explains that the microstructure of gluten becomes looser after fermentation and confirms that the large increase in FAA after fermentation is associated with changes in protein conformation. β-sheets are maintained by hydrogen bonds between peptide chains or between peptide chain segments. The increase in β-sheet content suggests that a large number of small molecule peptides are produced during microbial fermentation and that the peptide chains are reconnected by hydrogen bonds, leading to an increase in their content. β-turn content before and after fermentation there was no significant change, indicating that there was no significant change in the spatial conformation formed between the peptide chains of the protein molecules before and after fermentation. The random coils is an important region for the functional implementation and conformation of protein molecules, and its trend has an important impact on the properties of FWG. In conclusion, the structural changes can explain the side effects of the changes in the properties of FWG, such as its antioxidant properties.

### 3.3. Effect of fermentation on the amino acid composition of FWG

The FAA content is an important parameter for the degree of protein hydrolysis as well as the change in bioactivity after hydrolysis (33). The changes in FAA content during the fermentation process are shown in Table 1. Overall, the total free amino acid content showed an increasing trend with the extension of fermentation time, reaching its highest after 120 h of fermentation and significantly decreasing after 168 h of fermentation. It indicates that gluten powder is degraded by the action of *Bacillus subtilis*, releasing a large amount of FAA, among which Ala, Val, Ile, Leu, Met, Phe, Pro, and Gly are known as hydrophobic amino acids (HAA), whose hydrophobic groups can also act as intermediates of peptide-lipid interactions, affecting and regulating the biological activity of peptides (34). Kiyapey et al. (35) performed amino acid analysis of the obtained bioactive peptides with high antioxidant capacity and showed that their antioxidant capacity was significantly correlated with the HAA content. Zheng et al. (36) similarly found that the antioxidant activity of oat protein hydrolysate was positively correlated with the HAA content in it. This suggests that the high percentage of HAA plays a positive role in delaying lipid oxidation, which may be partly due to the fact that the exposure of HAA residues helps the peptide to bind to lipid radicals, thus enhancing the antioxidant activity of the hydrolysate. Therefore, we focused on the content of HAA in the fermentation products. As can be seen from Table 1, the HAA content in the gluten fermentation broth increased significantly (*p* < 0.05) with the increase in fermentation time, and the HAA content varied significantly at different times of fermentation. Accounting for 20.80, 75.56, 68.18, and 67.65% of the total amino acid at 24, 72, 120, and 168 h, respectively. The total HAA content reached a maximum of 1311.42 μg/mL after 120 h of fermentation, which was 129 times higher than the content at 24 h of fermentation. This indicates that fermentation significantly increased the content of HAA (*p* < 0.05).

**Table 1:** Changes of various amino acids at different fermentation times.

Sample fermentation time(h)	Total FAA (μg/mL)	BCAA (μg/mL)	AAA (μg/mL)	Ratio of BCAA (%)	Ratio of AAA (%)	Fischer’s ratio	HAA (μg/mL)	Ratio of HAA (%)
24	48.745 ± 0.049d	2.137 ± 0.007d	2.576 ± 0.007d	4.384 ± 0.013d	5.285 ± 0.009d	0.830 ± 0.003d	10.138 ± 0.011d	20.798 ± 0.015d
72	700.159 ± 0.390c	422.458 ± 0.087c	195.74 ± 0.031c	60.337 ± 0.036a	27.957 ± 0.020a	2.158 ± 0.001a	529.051 ± 0.066c	75.562 ± 0.046a
120	1923.381 ± 0.050a	770.876 ± 0.098a	483.351 ± 0.055a	40.079 ± 0.006b	25.130 ± 0.002b	1.595 ± 0c	1311.422 ± 0.130a	68.183 ± 0.008b
168	1710.083 ± 0.097b	651.082 ± 0.066b	403.518 ± 0.049b	38.073 ± 0.004c	23.596 ± 0.004c	1.614 ± 0b	1156.795 ± 0.065b	67.646 ± 0.001c

Results are reported as mean ± SD (*n* = 3), Values marked with different letters are significantly different at *p* < 0.05. FAA, Free amino acids; AAA, Aromatic amino acids (Phe, Trp, Tyr); BCAA, Branch chain amino acids (Leu, Ile, Val); HAA, Hydrophobic amino acids (Ala, Val, Ile, Leu, Met, Phe, Pro, Gly).

The BCAA are essential amino acids that play an irreplaceable role in the regulation of human physiology (37) and include three common amino acids: Leu, Val, and Ile. It has been shown to have various activities such as anti-aging (38), antioxidant (39), liver protection (40), and improving body functions (41). Studies have shown that supplementation with appropriate amounts of BCAA can increase serum antioxidant levels, reduce hepatic iron levels, decrease reactive oxygen species production, promote the expression of mitochondrial superoxide dismutase and improve the activity of mitochondrial complex I in the liver thereby slowing down oxidative stress and further inhibiting the development of hepatocellular carcinoma in patients with cirrhosis (42). Interestingly, different levels of BCAA play distinct roles in lipid oxidation and related metabolism (43), further experimental verification is necessary. In view of the important role of BCAA in ameliorating many diseases, the changes of Fischer ratio during gluten fermentation were monitored. Results as shown in Table 1, after 24 h of fermentation, the content of FAA in fermentation broth was only 48.74 μg/mL, among which BCAA were only 2.14 μg/mL, accounting for 4.4%. After 120 h of fermentation, the content of FAA in fermentation broth reached the highest value of 1923.38 μg/mL, among which BCAA accounted for the highest proportion, reaching 40.08% of the total FAA. The content of BCAA in the fermentation broth at 120 h was 360 times that at 24 h. At this time, the highest content of BCAA in the fermentation broth was observed. Therefore, after 120 h of fermentation, high Fischer ratio oligopeptides can be prepared by further treatment (activated carbon adsorption, gel filtration) of the fermentation broth to remove aromatic amino acids (AAA).

### 3.4. Changes in pH, soluble protein of fermentation broth during fermentation

**Figure 3:**
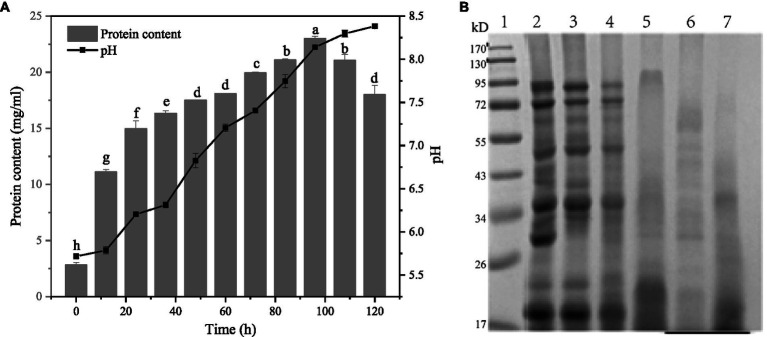
Changes of pH, soluble protein content and SDS-PAGE electrophoretic pattern of gluten of fermentation broth at different fermentation times. **(A)** Changes of pH, soluble protein content of fermentation broth at different fermentation times. **(B)** Changes of SDS-PAGE electrophoretic pattern of gluten in different fermentation stages of *Bacillus subtilis* fermentation. Lane 1: Marker, lane 2: unfermented gluten, lane 3–7 are gluten fermented for 0, 12, 24, 36, and 48 h.

Figure 3A shows the changes of pH and soluble protein content in the fermentation system at different fermentation times. In general, the pH and soluble protein content of the fermentation broth showed an increasing trend with the extension of the fermentation time, and the pH gradually increased from 5.71 at the beginning to 8.38 after 120 h of fermentation. However, it showed a decreasing trend after 108 h of fermentation. The change of soluble protein content in the fermentation system can be divided into two stages. The first stage was 0–60 h. In this stage, the soluble protein content changed rapidly with the fermentation time, increasing from the initial 2.81 mg/mL to 18.07 mg/mL, while in the second stage, the soluble protein content grew slowly during 60–108 h. The fermentation substrate was reduced by the fermentation substrate. It is possible that the enzyme activity of the fermentation system decreased with increasing pH due to the decrease in fermentation substrate and changes in enzyme activity in the fermentation broth that affected the protein degradation.

### 3.5. SDS-PAGE electrophoretogram

The SDS-PAGE electrophoretic pattern of the supernatant protein of fermentation broth at different fermentation times was shown in Figure 3B.

The first lane is the standard protein. According to the distribution of the electrophoretic pattern, the influence of fermentation on the gluten subunit can be clearly seen. The second lane on the left is unfermented gluten. It can be clearly seen that the band is relatively clear, the protein structure is complete, and the relative molecular weight is mainly distributed between 17 and 100 kDa, among which there are three obvious bands in the middle: 50, 35, and 30 kDa, in addition to two high molecular weight subunits containing 100 and 70 kDa and a low molecular weight subunit at 20 kDa.

By observing the protein spectra after gluten fermentation, it can be seen that fermentation has a significant effect on the change of molecular weight. Compared with wheat germ protein extracted at 12 h of fermentation and 0 h of fermentation, the bands at 100 and 70 kDa became lighter, and the bands around 17–25 kDa became darker, indicating that macromolecular proteins in fermentation broth depolymerized under the action of microorganisms, and the protein concentration decreased after 12 h of fermentation. With the prolongation of fermentation time, the large molecular weight protein gradually decreased, and the small molecular peptide gradually aggregated. The color of the bands in the range of 17–25 kDa gradually became darker, and the color of the bands in the range of 100, 70, 50, 35 kDa gradually became lighter and disappeared. *Bacillus subtilis* can synthesize extracellular protease itself, under the action of enzymes, gluten gradually degrades into small-molecule polypeptides, these small-molecule polypeptides have a variety of biological activities (44). Padhi et al. (11) fermented soybeans with *Bacillus subtilis* and identified 16 peptides by LC–MS/MS analysis, including the common peptide PFPIPFPIPIPLP and the characteristic peptide IPFPPIPFLIP, which had a significant effect on the inhibitory activity of angiotensin-converting enzyme (ACE). Karagiot et al. (45) isolated and purified the peptides produced by *Bacillus subtilis*, and compared them with UniProt and PubChem databases for identification. Bacteriocin Subtilosin-A (Q1W152), Subtilosin-Sbox (h6D9P4), Ericsson B (Q93Gh3), Subtilin (P10946) and several small-molecular peptides corresponding to non-ribosomal antimicrobial lipid peptide surface protein (CID:443592) were obtained. Compared with unfermented gluten, gluten has higher bioactivity after fermentation, which can be used in the development and production of small-molecule bioactive peptides, and is widely used in the biological health industry.

### 3.6. Free radical scavenging activity

**Figure 4:**
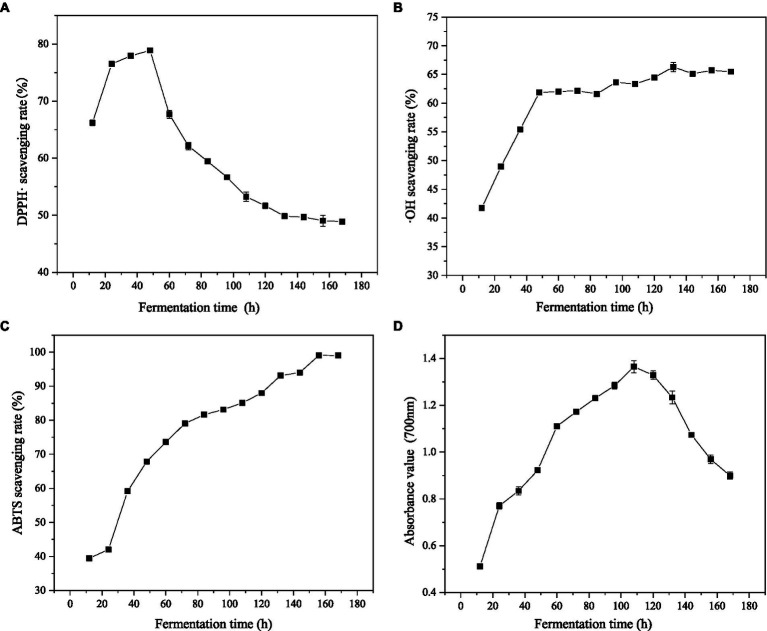
Effects of different fermentation time on DPPḤ, ·OH, ABTS scavenging rate and iron-reducing power. **(A)** Effects of different fermentation time on DPPḤ scavenging rate. **(B)** Effects of different fermentation time on ·OH scavenging rate. **(C)** Effects of different fermentation time on ABTS scavenging rate. **(D)** Effects of different fermentation time on iron-reducing power.

DPPḤ is a stable free radical. Its single electron can be paired with antioxidants to reduce the absorbance value of the reaction solution at 520 nm, which is widely used for the quantitative determination of antioxidant capacity. ABTS reacts with potassium perdisulphate to produce green ABTS radical. The free radical has its maximum absorption at 734 nm, so by detecting the absorbance at 734 nm, the antioxidant capacity of the tested substance can be determined (46). ·OH is mainly generated by the reaction of superoxide anion and hydrogen peroxide and is also the most active of all oxygen free radicals, which can cause serious damage to almost all adjacent oxygen free radicals (47). The scavenging activities of gluten fermentation products on DPPḤ, ·OH and ABTS free radicals were studied at different fermentation times (Figures 4A–C).

When the initial solid/liquid ratio was 1/10 (w/v), the scavenging rates of the three kinds of free radicals increased with the prolonging of fermentation time. After 48 h of fermentation, the scavenging rates of DPPḤ, ·OH and ABTS free radicals reached 78.89, 61.85, and 67.86%, respectively. At this time, the scavenging rate of DPPḤ radical reached the maximum value. With the continuing of fermentation, the scavenging rate of ·OH radical decreased, reaching the maximum value of 66.31% after 132 h of fermentation, while the scavenging rate of the ABTS radical showed a continuous increasing trend and began to decrease after reaching the maximum value of 99.08% at 168 h. The results of the three free radical scavenging rates show that the antioxidants in gluten can inhibit the formation of various free radicals or react with free radicals so as to break the free radical chain reaction and reduce the harm to organisms. The obvious increase in antioxidant activity after fermentation is closely related to the many active peptides and amino acids produced during fermentation. Many studies have shown that active peptides and FAA have antioxidant activities.

Zhang et al. (10) reported that the peanut peptide prepared by *Bacillus subtilis* fermentation of peanut meal had a maximum scavenging rate of 63.28% against DPPḤ at a concentration of 1.0 mg/mL. Li et al. (17) isolated a novel antioxidant peptide from chickpea protein hydrolysate, which had high DPPḤ and ·OH scavenging activities, 85.46 and 85.18%, respectively. In addition, the differences in antioxidant properties after fermentation may be related to qualitative and quantitative changes in the enzyme activities of strains, substrate species, and substrates. The intracellular antioxidants, peptides, and some metabolites secreted by the starter microorganisms, such as polysaccharides and phenolic compounds, may also be partly responsible for the increased antioxidant properties after fermentation. He et al. (25) showed that the antioxidant activity of soybean fermentation products was positively correlated with fermentation time and the concentration of total phenol and methanol extract, indicating that microbial fermentation played an important role in improving antioxidant activity. The fermented yeast rice product (FRYP) obtained from *Bacillus subtilis* fermentation by Gum et al. (48) had stronger antioxidant effects and free radical scavenging ability than before fermentation, which may be due to the high content of phenolic compounds and benzofuranone (one of the metabolites of transformation). However, the properties, structure–activity relationships and possible mechanisms of antioxidant compounds remain to be studied.

The antioxidant in FWG is able to reduce K_3_[Fe (CN)_6_] and then use ferrous ions to generate prussian blue, which has a maximum absorption peak at 700 nm. The higher absorbance value indicates the stronger reducing power of the sample. The reducing power of wheat gluten fermentation products at different fermentation times is shown in Figure 4D. Within 0–108 h, the reducing power gradually increased with the increase in fermentation time. At 108 h, the absorbance value at 700 nm was 1.35, which was 2.7 times higher than that before fermentation. After that, with the progress of hydrolysis, the absorbance value showed a downward trend, and the reducing power gradually decreased. This suggests that *Bacillus subtilis* fermentation of gluten powder changes its protein structure and releases some small-molecule active peptides and amino acids with specific electron donor capacities, demonstrating the reducing power. This is consistent with the results of previous studies. This indicates its potential application value as a natural reducing agent in food additives, medical care, and the cosmetic industries.

### 3.7. Thermo gravimetric analysis

**Figure 5:**
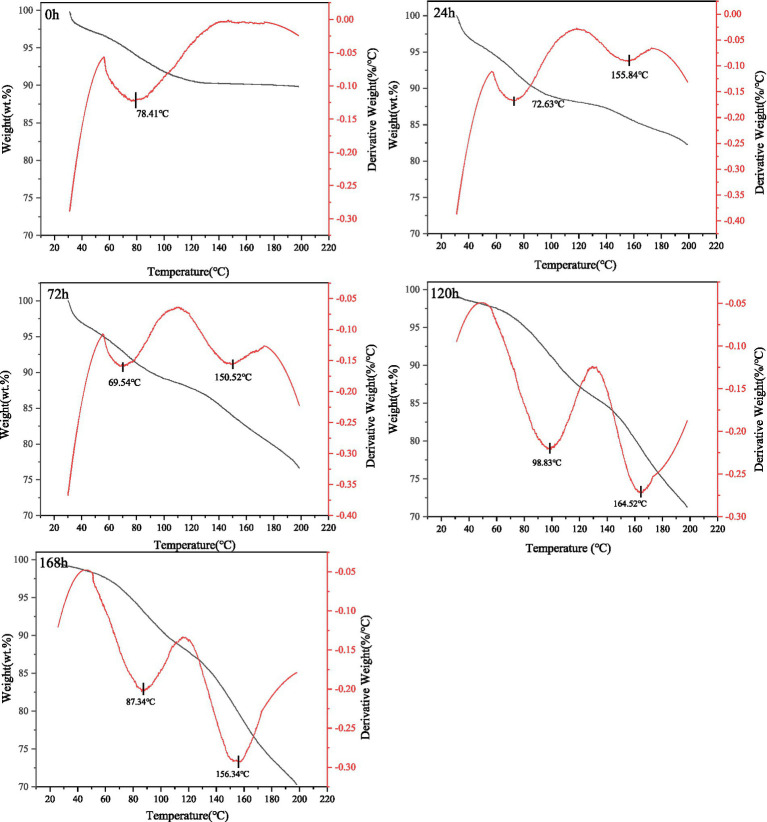
Thermo gravimetric analysis of fermentation substrate at different fermentation times.

Thermal degradation and stability are necessary considerations in industrial applications, and thermal treatment of protein and peptide products has been widely used in the food industry and protein-based materials. The unique protein composition and microstructure of gluten provides an excellent method to explain the structure-processing-property relationship. The effect of fermentation on the thermal stability of gluten was studied using thermo gravimetric analysis, and derivative thermogravimetrc (DTG) curves were made, with the physical significance of the DTG curves indicating the rate of weight loss as a function of temperature. The thermogravimetric changes of gluten before and after fermentation in the temperature range of 20–220°C are shown in Figure 5.

All samples showed a more pronounced first weight loss in the temperature range of 60–100°C, mainly due to evaporation of free water; the control’s first weight loss rate reached its maximum at 78.41°C. The maximum first weight loss of the samples at 24 and 72 h of fermentation corresponded to temperatures of 72.63°C and 69.54°C, respectively, both lower than the control (*p* < 0.05). As the fermentation time increases, the non-covalent and partially covalent bonds between the gluten molecules break, which causes its three-dimensional structure and protein network to become more loosely bound and less thermally stable from its initially tightly bound ordered and stable state (49). As a result, the second weight loss occurs much earlier after the temperature increase (155°C ≤ T ≤ 165°C). On the other hand, the absence of a second weight loss before 220°C in the control may be due to the fact that it retains its original structure, which consists mainly of a chain structure of gluten high molecular weight subunits and low molecular weight subunits cross-linked by interchain disulfide bonds and intramolecular disulfide and non-covalent bonds formed by alcoholic soluble proteins. Gliadin is present as a gap-filling cluster protein in the whole glutenin network structure γ-glutenin and low molecular weight glutenin (LMW-GS) polymerize during heat treatment, greatly increasing the stiffness and elasticity of glutenin. Thiol-rich lysins (α-, β-, and γ-glutenin) form hydrogen and disulfide bonds with LMW-GS, leading to polymerization and a firm tissue state, so that unfermented gluten is less thermally sensitive. Overall, the thermal stability of fermented gluten is reduced, which provides a reference for the post-processing and serving properties of fermented gluten.

## 4. Conclusion

*Bacillus subtilis* fermentation alters the microstructure and physicochemical properties of gluten proteins. The fermentation process resulted in the hydrolysis of gluten and a significant increase in FAA content, especially hydrophobic amino acids with antioxidant activity. Fermentation further changed the secondary structure of the WG, with a significant decrease in the proportion of random coil and a significant increase in the proportion of β-sheet in the FWG with increasing fermentation time (*p* < 0.05). SDS-PAGE results further demonstrated the ability of *Bacillus subtilis* to degrade gluten proteins and release small-molecule active peptides. In addition, the antioxidant activity of FWG was significantly increased compared to WG; the change in antioxidant activity could be attributed to the increased content of various peptides. Fermentation with *Bacillus subtilis* is an effective method to increase the proteolytic and antioxidant activity of WG, and FWG can be used as a functional ingredient to create new foods. This paper provides a theoretical framework for further investigation of the FWG’s functional properties. However, the isolation and characterization of bioactive small molecule peptides, antioxidant compounds and physiological factors secreted by microorganisms produced in fermentation, as well as their conformation-activity relationships and possible mechanisms remain to be investigated.

## Data availability statement

The original contributions presented in the study are included in the article/supplementary material, further inquiries can be directed to the corresponding author.

## Author contributions

P-hZ: conceptualization, methodology, software, and writing – original draft preparation. Y-CH: conceptualization, writing – reviewing and editing, and supervision. ZW: methodology, writing – reviewing and editing, software, and visualization. A-ML and LP: supervision and writing assistance. JZ, Y-QD, Z-YH, and X-QO: language and software. J-HH: funding acquisition, supervision, and project administration. All authors contributed to the article and approved the submitted version.

## Funding

The authors would like to thank Major Science and Technology Projects for Public Welfare of Henan Province (No: 201300110300), Innovation Demonstration Special Project of Henan Province (No: 201111110100), Zhongyuan Scholars of Henan Province in China (No: 192101510004), Zhongyuan Scholar Workstation Funded Project (Nos: ZYGZZ2020015 and 214400510015), Henan Provincial Key Science & Technology Special Project (No: 221100110700), and Central Government Guides the Local Science and Technology Development Special Fund (No: Z20221341069) for funding this research.

## Conflict of interest

The authors declare that the research was conducted in the absence of any commercial or financial relationships that could be construed as a potential conflict of interest.

## Publisher’s note

All claims expressed in this article are solely those of the authors and do not necessarily represent those of their affiliated organizations, or those of the publisher, the editors and the reviewers. Any product that may be evaluated in this article, or claim that may be made by its manufacturer, is not guaranteed or endorsed by the publisher.

## Abbreviations

SEM: Scanning electron microscopy
FT-IR: Fourier transformed infrared spectroscopy
CD: Circular dichrography
SDS-PAGE: Sodium dodecyl sulfate-polyacrylamide gel electrophoresis
DPPḤ: 1,1-Diphenyl-2-picrylhydrazyl radical 2,2-Diphenyl-1-(2,4,6-trinitrophenyl) hydrazyl
·OH: Hydroxyl radical
ABTS: 2,2′-Biazo-bis-3-ethylbenzothiazoline-6-sulfonic acid
WG: Wheat gluten
BCA: Bicin-choninic acid
FWG: Fermented wheat gluten
FAA: Free amino acids
BCAA: Branched-chain amino acids
HAA: Hydrophobic amino acids
AAA: Aromatic amino acids
TCA: Trichloroacetic acid
TGA: Thermo gravimetric analysis
DTG: Derivative thermogravimetrc
LMW-GS: Low molecular weight glutenin
